# Downregulating Long Non-coding RNAs CTBP1-AS2 Inhibits Colorectal Cancer Development by Modulating the miR-93-5p/TGF-β/SMAD2/3 Pathway

**DOI:** 10.3389/fonc.2021.626620

**Published:** 2021-04-14

**Authors:** Qiankun Li, Wenjing Yue, Ming Li, Zhipeng Jiang, Zehui Hou, Wei Liu, Ning Ma, Wenchang Gan, Yingru Li, Taicheng Zhou, Wenjing Yue, Shuang Chen

**Affiliations:** ^1^Department of Gastroenterology, Yantai Affiliated Hospital of Binzhou Medical University, Yantai, China; ^2^Guangdong Provincial Key Laboratory of Colorectal and Pelvic Floor Diseases, Supported by National Key Clinical Discipline, Department of Gastrointestinal Surgery, Guangdong Institute of Gastroenterology, The Sixth Affiliated Hospital of Sun Yat-sen University, Guangzhou, China

**Keywords:** CRC, progression, CTBP1-AS2, TGF-β/SMAD2/3 pathways, MiR-93-5p

## Abstract

**Background:** Colorectal cancer (CRC), the most commonly diagnosed cancer in the world, has a high mortality rate. In recent decades, long non-coding RNAs (lncRNAs) have been proven to exert an important effect on CRC growth. However, the CTBP1-AS2 expression and function in CRC are largely unknown.

**Materials and Methods:** The CTBP1-AS2 and miR-93-5p expression in CRC and para-cancerous tissues was detected by reverse transcription-PCR. The expression of CTBP1-AS2, miR-93-5p and the transforming growth factor-beta (TGF-β)/small mothers against decapentaplegic 2/3 (SMAD2/3) pathway was selectively regulated to study the correlation between CTBP1-AS2 expression and prognosis of patients with CRC. CRC cell proliferation, apoptosis, and invasion were measured *in vivo* and *in vitro*. In addition, bioinformatics was applied to explore the targeting relationship between CTBP1-AS2 and miR-93-5p. The targeting binding sites between CTBP1-AS2 and miR-93-5p, as well as between miR-93-5p and TGF-β, were verified by the dual-luciferase reporter assay and the RNA immunoprecipitation experiment.

**Results:** Compared with normal para-cancerous tissues, CTBP1-AS2 was considerably overexpressed in CRC tissues and was closely associated with worse survival of patients with CRC. Functionally, gain and loss in experiments illustrated that CTBP1-AS2 accelerated CRC cell proliferation and invasion and inhibited cell apoptosis. Mechanistically, CTBP1-AS2 regulated the malignant phenotype of tumor cells through the TGF-β/SMAD2/3 pathway. Moreover, miR-93-5p, as an endogenous competitive RNA of CTBP1-AS2, attenuated the oncogenic effects mediated by CTBP1-AS2.

**Conclusion:** CTBP1-AS2 promotes the TGF-β/SMAD2/3 pathway activation by inhibiting miR-93-5p, thereby accelerating CRC development.

## Introduction

Worldwide, colorectal cancer (CRC) is the third most prevalent malignant tumor in both men and women and is the third most common factor for tumor-related deaths (1). With the continuous progress of hospital diagnosis and treatment, the 5-year survival period of patients with CRC is extending (2). However, early diagnosis is difficult due to its occult onset. Therefore, it is crucial to explore the early molecular diagnostic indicators of CRC.

Long non-coding RNAs (lncRNAs) are >200 nucleotides in length. Functionally, lncRNAs affect gene expression through various mechanisms such as silencing X chromosome, chromatin modification, imprinting genome, transcriptional activation, transcriptional interference, and nuclear transport (3, 4). In particular, a variety of lncRNAs are aberrantly expressed in CRC and modulate its malignant phenotypes. Taking lncRNA GAS8-AS as an example, it was downregulated in CRC, and it inhibited CRC cell proliferation by inhibiting the expression of AFAP1-AS1, another lncRNA with carcinogenic effects (5). In another study, lncRNA NEAT1 indirectly activated the Wnt/β-catenin signaling pathway through DDX5, thereby promoting CRC cell proliferation, migration, and invasion (6). CTBP1-AS2 is 2945 bp long and is located at 4p16.3. Previous studies have stated that CTBP1-AS2 can be used as a diagnostic indicator for type 2 diabetes (7). Besides, CTBP1-AS2 stabilizes toll-like receptor 4 (TLR4) and regulates cardiomyocyte hypertrophy by interacting with FUS RNA binding protein (8). Here, we have testified that CTBP1-AS2 has increased high expression in healthy human tissues (Supplementary Figure 1A), and its expression is upregulated in a variety of tumor tissues. However, its effect on CRC development is limited.

Together with lncRNAs, microRNAs (miRNAs) are a class of small RNAs with 18–24 bp in length. Various studies have discovered that miRNAs regulate tumor growth (9). As a miRNA, miR-93-5p inhibits the Hippo signaling pathway, thus promoting gastric cancer cell process (10), and its knockdown inhibits CRC cell proliferation and metastasis by targeting programmed death ligand 1 (PD-L1) (11). Functionally, miRNAs are competitively inhibited by lncRNAs, on the other hand, it binds to the 3′ untranslated region (3′-UTR) of mRNAs. This lncRNA–miRNA-mRNA network is believed to contribute significantly to the regulation of tumor progression (12).

Here, we detected CTBP1-AS2 expression in CRC tissues. The results revealed that CTBP1-AS2 expression was notably increased in CRC tissues and cells, and it was closely related to the worse survival of patients with CRC. In terms of the functions, CTBP1-AS2 upregulation promoted CRC cell proliferation, invasion, and inhibited cell apoptosis. Besides, CTBP1-AS2 significantly promoted the activation of the transforming growth factor-β (TGF-β)/small mothers against decapentaplegic 2/3 (SMAD2/3) signaling pathway. Furthermore, through bioinformatics analysis, we found that miR-93-5p was a targeted miRNA for CTBP1-AS2 and TGF-β. Therefore, we hypothesized that there was a key regulatory axis in CRC, namely, the CTBP1-AS2-miR-93-5p-TGF-β/SMAD2/3 network.

## Materials and Methods

### Organization and Samples

From July 2014 to March 2015, 50 CRC tumor tissues and their paired healthy tissues were obtained from the Sixth Affiliated Hospital of Sun Yat-sen University. The adjacent healthy tissues were >3 cm away from the tumor edge and were stored in a refrigerator at −80°C, once isolated. All patients received surgical treatment in the Sixth Affiliated Hospital of Sun Yat-sen University and were independently diagnosed as having CRC by two pathologists. None of the selected patients received radiotherapy or chemotherapy before the operation. The general clinical data such as age, sex, height, weight, and tumor characteristics were collected, and the patients' prognosis was followed by telephone. This research was approved by the ethics committee of the Sixth Affiliated Hospital of Sun Yat-sen University. All patients gave their written informed consent prior to participation.

### Cell Culture

Human intestinal mucosal normal cell fetal human cell (FHC) and CRC cell lines SW620, HCT116, SW480, T84, Caco-2, and HT29 were purchased from the American Type Collection Center. All of the cells were cultured in the Roswell Park Memorial Institute Medium (RPMI) 1640 medium (Thermo Fisher Scientific, MA, USA), which contained 10% fetal bovine serum (FBS) (Thermo Fisher Scientific, MA, USA) and 1% penicillin/streptomycin (Invitrogen, CA, USA). The medium was placed in an incubator with 5% CO_2_ at 37°C, and the solution was changed every 2–3 days. During the logarithmic growth period, the cells underwent 0.25% trypsinization (Thermo Fisher HyClone, UT, USA) for subculture.

### Cell Transfection and Treatment

The pcDNA3.1 empty vector (pcDNA3.1), pcDNA-CTBP1-AS2 (CTBP1-AS2), small interfering RNA negative control (si-NC), siRNA against CTBP1-AS2 (si-CTBP1-AS2), miRNA negative control (miR-NC), and miR-93-5p mimics were purchased from GenePharma Co. Ltd. (Shanghai, China). SW620 and HT29 cells were inoculated in 24-well plates (3 × 10^5^ cells per well), incubated for 24 h at 37°C in an incubator with 5% CO_2_, and transfected with the above-mentioned vectors using Lipofectamine 3000 (Invitrogen; Thermo Fisher Scientific, Shanghai, China) according to the specifications of the manufacturer. The cells were then cultured for 24 h at 37°C in an incubator with 5% CO_2_. Finally, the original medium was replaced with a complete fresh medium. After 24 h of culture, the transfection efficiency was detected by reverse transcription (RT)-PCR.

Recombinant human transforming growth factor beta 1 (TGF-β1, catalog no. PRP100188) was purchased from Wuhan AmyJet Scientific Inc. LY2109761 (catalog no s2704) was a new, selective TGF-β receptor type I/II dual inhibitor, and was purchased from Selleck (Shanghai, China). When the cells were in the stable growth stage, TGF-β1 (100 ng/ml) and LY2109761 (5 μM) were adopted to treat the cells for 4 h.

### Cell Counting Kit-8 Assay

SW620 and HT29 cells in the exponential growth phase were taken and made into a single-cell suspension. After cell counting, cell density was adjusted (1,000 cells per well). They were then inoculated into 96-well plates (six replicates in each group, six plates in total). After 24, 48, and 72 h, we added 90 μl medium and 10 μl cell counting kit-8 **(**CCK-8) solution to the samples. Meanwhile, a blank control well-containing only medium and the CCK-8 solution was set. After 2 h of incubation, the absorbance (*A*) value of each well was measured and recorded with a microplate reader at 450 nm wavelength. The measurement was performed every 24 h for 5 days. The *A* value of the corresponding blank group was subtracted from the *A* value of each experimental group, and the cell proliferation in each group was calculated according to the standard curve.

### Colony Formation Experiment

The single-cell suspension was prepared from SW620 and HT29 cells and corresponding control cells in the logarithmic growth period. The cell suspension was diluted (50 cells/ml), and 10 ml cell suspensions were evenly inoculated in a 60 mm Petri dish and cultured in an incubator for 2–3 weeks. When the cells in the Petri dish formed visible colonies, we stopped the culture and washed the cells with phosphate-buffered saline (PBS) three times (3 min each time). The fixing solution was removed after 15 min of methanol fixing, and the excess dye solution was washed away after Giemsa staining solution was added for 30 min. Finally, the plate was inverted, and the number of colonies (10 cells/colony) was labeled under the microscope.

### Dual-Luciferase Reporter Assay

The sequences of CTBP1-AS2 and TGF-β1 were amplified and transfected into the downstream luciferase gene of the pMIRGLO plasmid (Promega, Fitchburg, WI, USA) to obtain pMIR-CTBP1-AS2-WT and pMIR-TGF-β1-WT. According to the binding sites between miR-93-5p and CTBP1-AS2 and TGF-β1, the fragments containing the mutant target region were designed as pMIR-CTBP1-AS2-MT and pMIR-TGF-β1-MT. CTBP1-AS2-WT, TGF-β1-WT, CTBP1-AS2-MT, and TGF-β1-MT were co-transfected with miR-NC or miR-93-5p mimics into SW620 cells using Lipofectamine 3000. After 48 h, the dual-luciferase reporter assay system was used to evaluate the luciferase activity.

### RNA Immunoprecipitation

SW620 cells were cultured in a T75 culture flask. When the cells were in the logarithmic growth stage and reached 80% fusion rate, they were trypsinized and centrifuged. Next, the cells were fully lysed with the complete radioimmunoprecipitation assay (RIPA) lysis buffer (Merck Millipore, MA, USA). After the supplementation of 100 μl cell lysates, the human anti-Ago2 antibody (Merck Millipore, MA, USA) and healthy mouse immunoglobulin G (IgG; Merck Millipore, MA, USA) were incubated overnight with rotation at 4°C. Afterward, RNA was eluted from the beads through 2-h incubation with 400 μl elution buffer. Next, the eluted RNA was precipitated with ethanol and dissolved with RNase-free water. The enrichment of certain fragments was detected by RT-PCR.

### Transwell Assay

SW620 and HT29 cells went through dispersing with 0.25% trypsin, centrifugation, and resuscitation. Transwell chambers (8 μm pore size; Corning, Beijing, China) were coated with BD Matrigel (Shanghai, China) overnight. We placed 5 × 10^4^ cells in the upper chamber and 10% FBS medium in the lower chamber, which was filled with 400 μl RPMI 1640. After 24-h incubation at 37°C, the non-invasive cells were removed from the upper chamber. The Transwell membranes were fixed with 4% paraformaldehyde (10 min) and then stained with 0.5% crystal violet. Then, the cells were counted under an inverted microscope after rinsing under running water. In this research, all the experiments were carried out in triplicate.

### Enzyme-Linked Immunosorbent Assay

After cell treatment, the medium was collected. SW620 and HT29 cells underwent 10-min centrifugation (1,000 rpm). The supernatant was taken, and the contents of TGF-β1 were examined according to the instructions of the manufacturer of the TGF-β1 kit (Abcam, ab100647, Shanghai, China).

### Reverse Transcription-PCR

Cells were cultured in six-well plates, trypsinized with 0.25% trypsin and centrifuged. Then, total RNA was extracted according to the instructions of the manufacturer of TRIzol reagent (Invitrogen, Shanghai, China), and its concentration and purity were detected by Thermo Fisher Scientific, Waltham, Massachusetts, USA. ReverTra Ace qPCR RT Kit (TOYOBO, Osaka, Japan) was used to reverse transcribe the total RNA into complementary DNA (cDNA). The cDNA obtained was used for RT-PCR using SYBR Green qPCR Master Mix (MedChemExpress, NJ, USA). The reaction conditions were set as follows: pre-denaturation at 95°C for 30 s, denaturation at 95°C for 5 s, and annealing at 60°C for 30 s, with 45 cycles in total. The target genes and endogenous control genes of each sample were amplified. Three duplicate wells were designed for each cell group. Meanwhile, the relative expression of RNA was verified by glyceraldehyde 3-phosphate dehydrogenase (GAPDH) or U6 gene-level correction and analyzed with the 2^−ΔΔCT^ method. The primer sequence was as follows: CTBP1-AS2, forward primer 5′-CGTTCTGATTCCTGGCATGG-3′, reverse primer 5′-TACCTCATCGACGTTCCCAG-3′; miR-93-5p, forward primer 5′- AACAATCAAAGTGCTGTTCGTGC-3′, reverse primer 5′- CAGTGCAGGGTCCGAGGT-3′.

### Western Blotting

After the treated cells were extracted, RIPA lysis buffer (Applygen Technologies, Beijing, China) was added. Next, the cells were fully lysed on ice and centrifuged at 14,000 rpm for 15 min at 4°C. After the supernatant was collected, the protein concentration was detected using the BCA kit (Thermo Fisher Scientific, Shanghai, China). Then, the protein was isolated by adding sodium dodecyl sulfate-polyacrylamide gel electrophoresis sample loading buffer (5X) (Beyotime, Wuhan, China). The separated proteins were later transferred to the polyvinylidene fluoride membranes. The membranes were sealed with 5% skimmed milk powder (room temperature, 1 h) and incubated with the addition of a primary antibody diluted with 5% skimmed milk, including anti-TGF-β1 antibody (Abcam, ab92486), anti-Smad2 (phospho S467) antibody (Abcam, ab53100), anti-Smad3 (phospho S423+S425) antibody (Abcam, ab52903), anti-Smad2 antibody (Abcam, ab40855), anti-Smad3 antibody (Abcam, ab40854), anticaspase 3 antibody (Proteintech, 19677-1-AP), and anti-Bax antibody (Proteintech, 50599-2-Ig) at 4°C overnight. The next morning, the membranes were rinsed with Tris-buffered saline with Tween 20 (TBST) (three times, 10 min each), coated with horseradish peroxidase-conjugated AffiniPure secondary antibody (2 h, room temperature). Afterward, the membranes were rewashed three times with TBST (10 min each). Then, the BeyoECL Plus Detection Kit (Beyotime, Wuhan, China) was used to expose the membranes, and the strip was displayed on an x-ray film. This study used β-actin as an endogenous control for the protein, and all trials were repeated three times.

### Tumor Formation Assay in Nude Mice

A total number of 20 female nude BALB/c mice aged 6 weeks were purchased from the Beijing Vital River Laboratory (Beijing, China). All animal procedures were performed in accordance with the protocols approved by the Animal Care and Use Committees of Yantai Affiliated Hospital of Binzhou Medical University. For the construction of the xenograft model, SW620 cells with stable transfection of CTBP1-AS2 overexpression vector or negative vector were harvested. Then, 1 × 10^6^ SW620 cells were suspended in 0.1 ml PBS and were injected subcutaneously in the right flank of the mice (10 mice per group). Tumor volumes were examined every 7 days when the implantations started to grow bigger. Tumor volumes were calculated by using the equation V (mm^3^) = A × B^2^/2, where A is the largest diameter and B is the perpendicular diameter. After 5 weeks of this experiment, the mice were sacrificed to collect the tumor tissues. The tumor tissues were weighed, and the expression of TGF-β1, Ki-67, terminal deoxynucleotidyl transferase-mediated dUTP nick-end labeling (TUNEL) was detected by immunohistochemistry (13) and/or western blotting. Moreover, the lung tissues of each mouse were isolated, and the lung metastasis of tumor cells was evaluated by H&E staining.

### Data Analysis

The data of this study were shown as mean ± SD using SPSS version 17.0 (SPSS Inc., Chicago, IL, USA) for data difference analysis. One-way ANOVA was performed for intergroup comparison. When the mean value of the groups was different, Student's *t-*test was employed for pairwise comparison between the groups. The percentage (%) or statistical data were compared using the χ^2^ analysis. Pearson's linear regression was taken to analyze the correlation between CTBP1-AS2 and miR-93-5p in the CRC tissues. The value of *p* < 0.05 was considered to be statistically significant.

## Results

### CTBP1-AS2 Was Upregulated in CRC Tissues and Was Associated With Worse Survival of Patients With CRC

**Figure 1:**
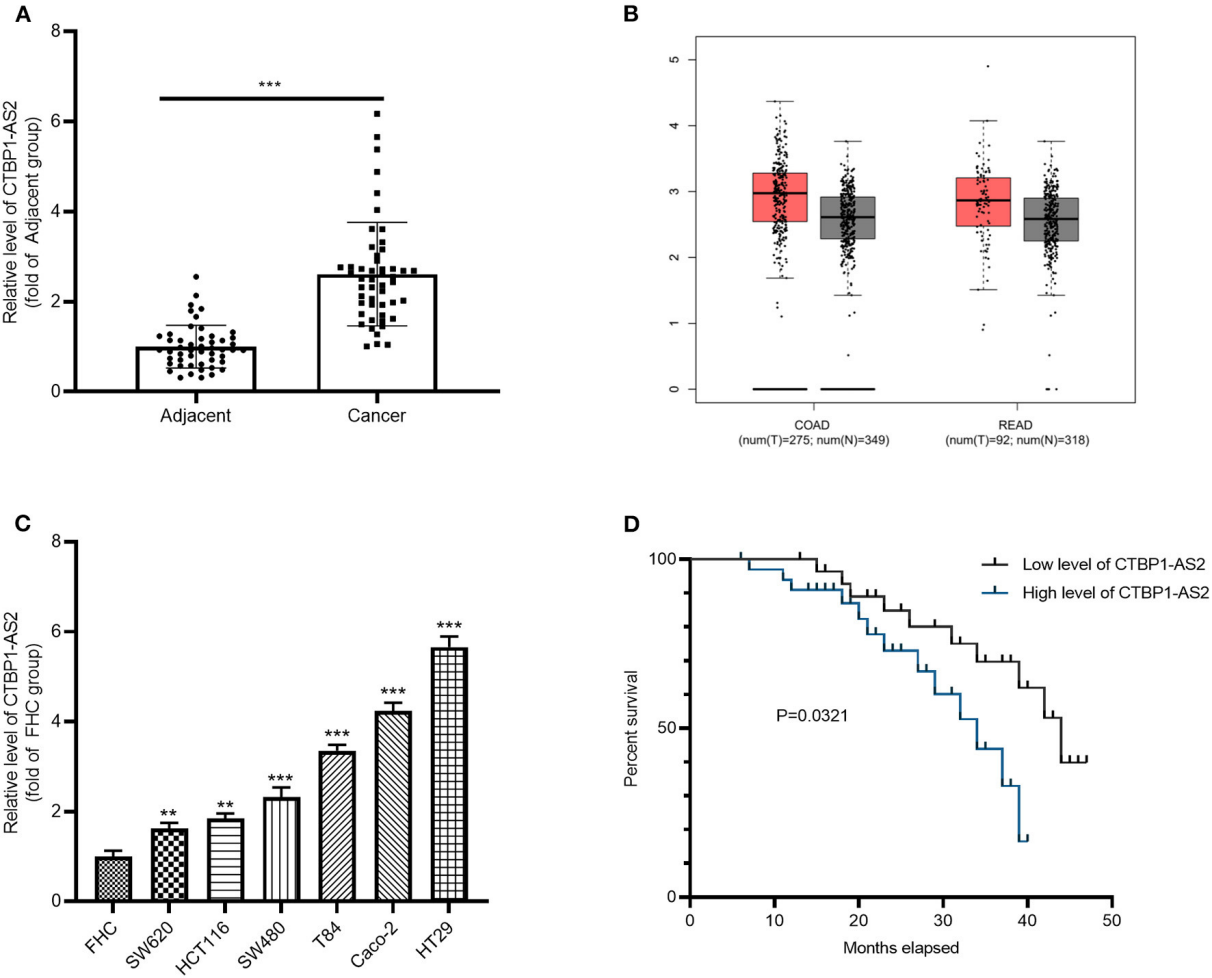
Increased expression of CTBP1-AS2 in CRC tissues and correlation with poorer prognosis in patients with CRC. **(A)** Use of RT-PCR to measure CTBP1-AS2 expression in 50 CRC tissues and adjacent healthy tissues, ***p < 0.001. **(B)** Analysis of CTBP1-AS2 expression in COAD and READ tissues by GEPIA. **(C)** Evaluation of CTBP1-AS2 levels in healthy human intestinal mucosa FHC and CRC cancer cell lines SW620, HCT116, SW480, T84, Caco-2, and HT29 by RT-PCR, **p < 0.01, ***p < 0.001 vs. FHC group. *N* = 3. **(D)**. Kaplan–Meier analysis of the association between CTBP1-AS2 level and the survival in patients with CRC. CRC, colorectal cancer; COAD, colon adenocarcinoma; FHC, fetal human cell; GEPIA, Gene Expression Profiling Interactive Analysis; READ, rectal adenocarcinoma; RT, reverse transcription.

To investigate the effect of CTBP1-AS2 in CRC, RT-PCR was employed to evaluate CRC expression in healthy tissues and para-cancerous tissues from 50 cases. It was found that CTBP1-AS2 was overexpressed in CRC as compared with normal adjacent tissues (Figure 1A). Through the Gene Expression Profiling Interactive Analysis (GEPIA) (http://gepia.cancer-pku.cn/), a newly developed interactive web server for analyzing the RNA-sequencing expression data of 9,736 tumors and 8,587 healthy samples from the The Cancer Genome Atlas and the Genotype Tissue Expression projects (14), we found that CTBP1-AS2 upregulation also occurred in most tumor tissues (Supplementary Figure 1B), including colon adenocarcinoma (COAD) and rectal adenocarcinoma (READ) (Figure 1B). Besides, we also found that CTBP1-AS2 expressions in CRC cancer lines SW620, HCT116, SW480, T84, Caco-2, and HT29 were significantly upregulated compared with normal human intestinal mucosal cells FHC (Figure 1C). Furthermore, we analyzed the correlation between CTBP1-AS2 level and prognosis of patients with CRC. The results revealed that those patients with CRC with higher expression of CTBP1-AS2 not only had a worse overall survival rate (Figure 1D), but also had larger tumor volumes and earlier distant metastasis (Table 1). These results suggest that CTBP1-AS2 can not only be used as a diagnostic indicator for CRC prognosis, but may also be involved in CRC development.

**Table 1:** Relationship between CTBP1-AS2 level and clinical characteristics in colorectal cancer.

**Indicators**		**CTBP1-AS2 level**		***P*-value**
		**High (*n* = 25)**	**Low (*n* = 25)**	
Age (years)	>50	18	20	0.5078
	≤ 50	7	5	
Gender	Male	16	14	0.5637
	Female	9	11	
Tumor stage	I–II	16	10	0.0894
	III–IV	9	15	
Tumor size (cm)	>5	8	15	0.047
	≤ 5	17	10	
Grade	High	16	11	0.1556
	Low	9	14	
Remote metastasis	Yes	7	14	0.0449
	No	18	11	

### The Regulation of CTBP1-AS2 on CRC Cell Proliferation, Apoptosis, and Invasion

**Figure 2:**
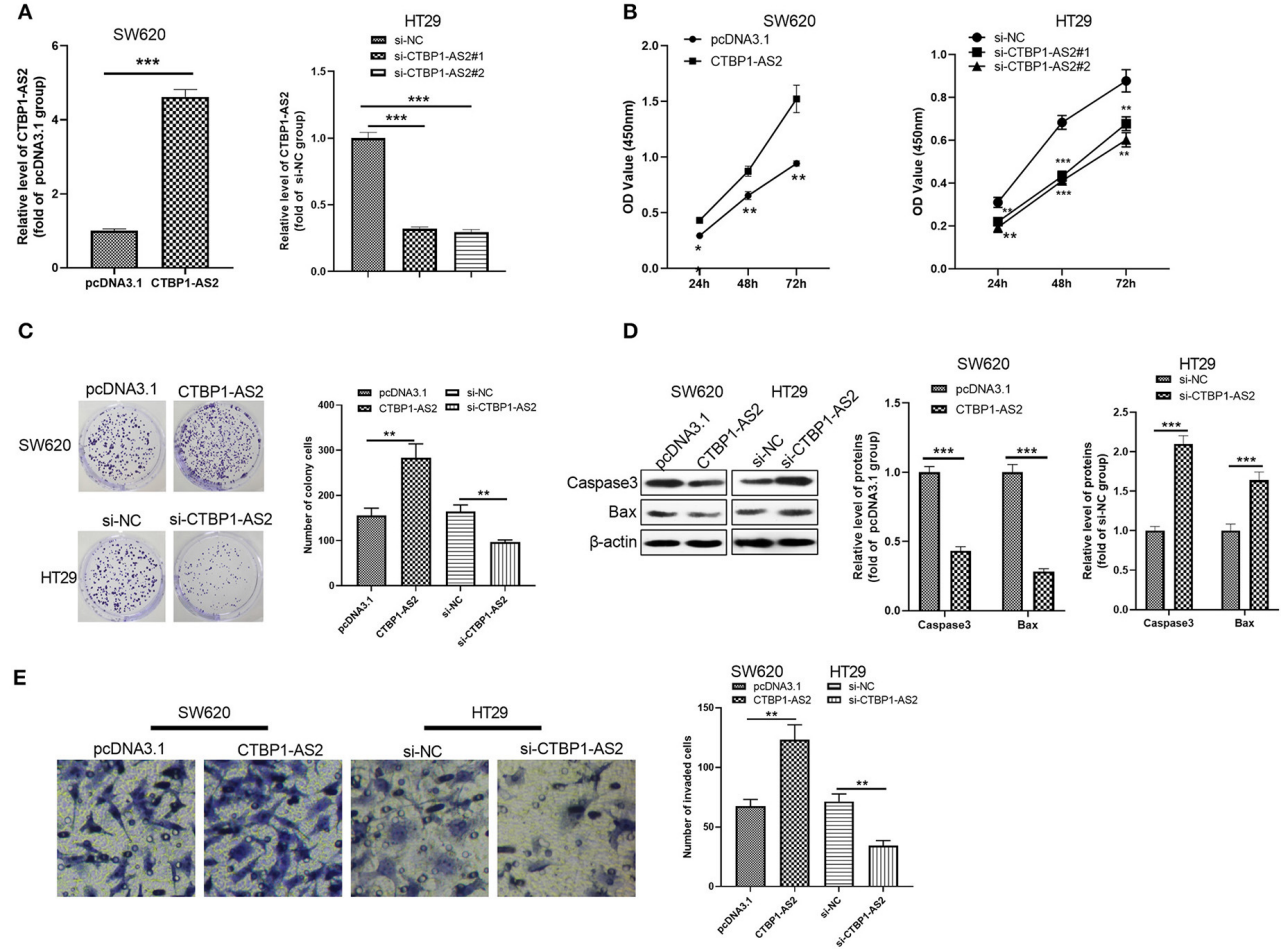
Regulation of CTBP1-AS2 on CRC proliferation, apoptosis, and invasion. **(A)** SW620 cells transfected with CTBP1-AS2 overexpression plasmids or pcDNA3.1, and HT29 cells transfected with si-CTBP1-AS2 or si-NC. Performing RT-PCT to measure CTBP1-AS2 expression in the two cell lines. **(B)** Incubation of SW620 and HT29 cells in the 96-well plates for different time points (24, 48, and 72 h). Use of CCK-8 assay to examine cell proliferation with different levels of CTBP1-AS2. *p < 0.05, **p < 0.01, ***p < 0.001 vs. pcDNA3.1 group or si-NC group. **(C)** Examination of cell proliferation of SW620 and HT29 cells through colony formation experiments. **(D)** Detection of apoptosis-related proteins caspase-3 and Bax expressions in SW620 and HT29 cells using Western blotting. **(E)** Use of the Transwell method to measure cell invasion of SW620 and HT29 cells. **p < 0.01, ***p < 0.001. *N* = 3. CCK-8, cell counting kit-8; CRC, colorectal cancer; RT, reverse transcription; si-NC, small interfering RNA negative control.

To further explore the effect of CTBP1-AS2 in CRC growth, we constructed a cell model of CTBP1-AS2 overexpression or knockdown (Figure 2A). CCK-8 and colony formation experiments were used to measure cell proliferation, and the results illustrated that the proliferative ability of SW620 cells with overexpressed CTBP1-AS2 was notably enhanced, while that of CTBP1-AS2 knockdown cells was considerably decreased (Figures 2B,C). Additionally, we applied Western blotting to examine the expression of apoptosis-related proteins caspase 3 and Bax in the tumor cells. The results showed that caspase 3 and Bax expressions were remarkably downregulated after the CTBP1-AS2 overexpression, while CTBP1-AS2 knockdown promoted their expressions (Figure 2D). Furthermore, cell invasion was detected by the Transwell method, and it was found that CTBP1-AS2 overexpression promoted CRC cell invasion, while CTBP1-AS2 knockdown inhibited cell invasion. Thus, CTBP1-AS2 upregulation accelerated CRC growth by promoting cell proliferation and invasion and inhibiting apoptosis.

### CTBP1-AS2 Activated TGF-β/SMAD2/3 Pathway in CRC Cells

**Figure 3:**
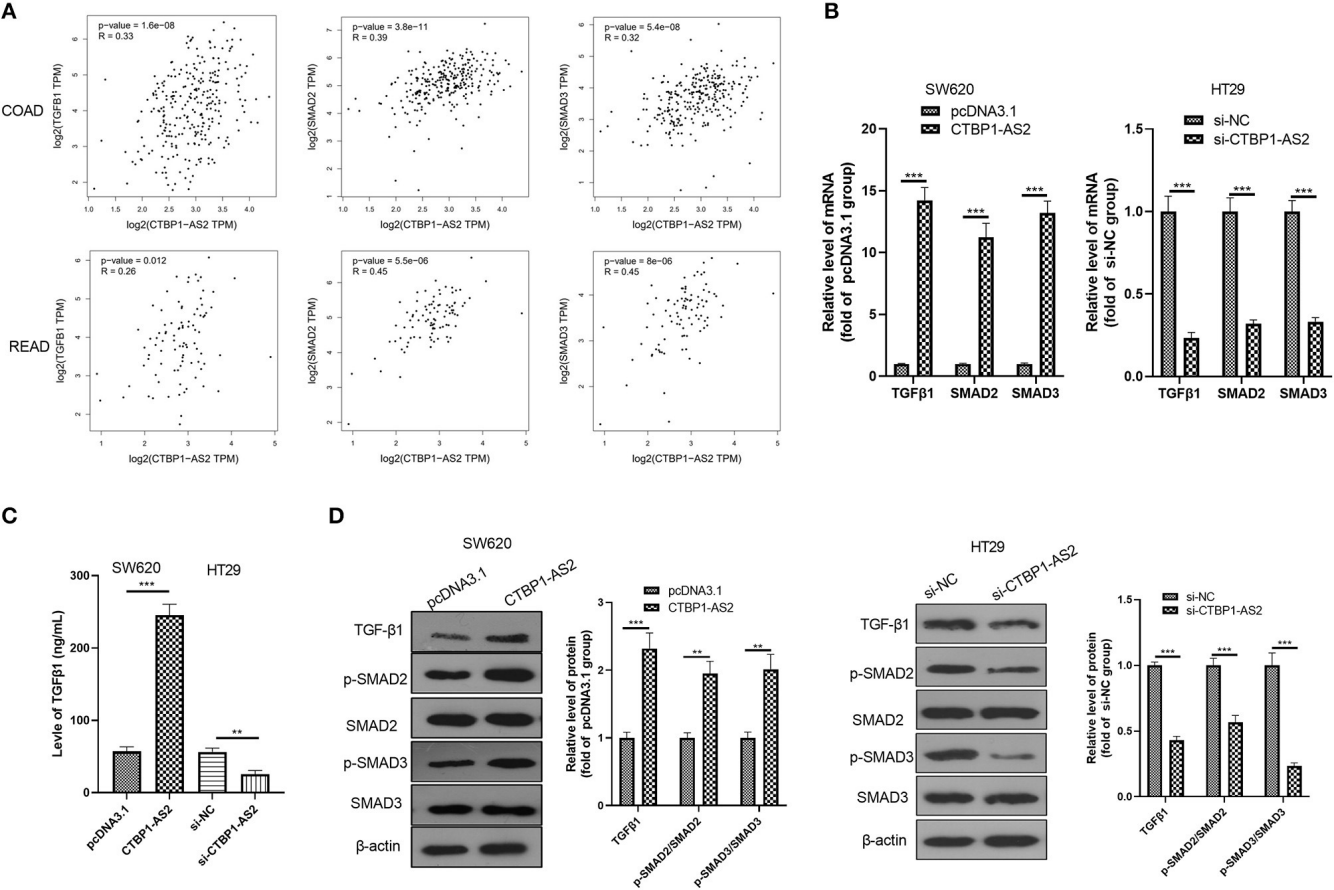
Regulation of CTBP1-AS2 on CRC proliferation, apoptosis, and invasion. **(A)** SW620 cells transfected with CTBP1-AS2 overexpression plasmids or pcDNA3.1, and HT29 cells transfected with si-CTBP1-AS2 or si-NC. Performing RT-PCT to measure CTBP1-AS2 expression in the two cell lines. **(B)** Incubation of SW620 and HT29 cells in the 96-well plates for different time points (24, 48, and 72 h). Use of CCK-8 assay to examine cell proliferation with different levels of CTBP1-AS2. *p < 0.05, **p < 0.01, ***p < 0.001 vs. pcDNA3.1 group or si-NC group. **(C)** Examination of cell proliferation of SW620 and HT29 cells through colony formation experiments. **(D)** Detection of apoptosis-related proteins caspase-3 and Bax expressions in SW620 and HT29 cells using Western blotting. **(E)** Use of the Transwell method to measure cell invasion of SW620 and HT29 cells. **p < 0.01, ***p < 0.001. *N* = 3. CCK-8, cell counting kit-8; CRC, colorectal cancer; RT, reverse transcription; si-NC, small interfering RNA negative control.

Through GEPIA database, we found that in both COAD and READ, CTBP1-AS2 expression level positively correlated with the level of TGF-β1, SMAD2, and SMAD3 (*p* < 0.01, Figure 3A). In view of this fact, we performed RT-PCR, ELISA, and Western blotting to detect TGF-β1, SMAD2, and SMAD3 levels in overexpressed and low-expressed CTBP1-AS2 cells. It was found that overexpressed CTBP1-AS2 promoted the expressions of TGF-β1, SMAD2, and SMAD3, as well as the phosphorylated levels of SMAD2/3, while CTBP1-AS2 knockdown had the opposite effect (Figures 3B–D). These statistics indicated that the TGF-β1/SMAD2/3 pathway is an important signaling pathway of CTBP1-AS2 downstream.

### The Intervention of TGF-β1/SMAD2/3 Pathway Reversed CTBP1-AS2-Mediated Effects

**Figure 4:**
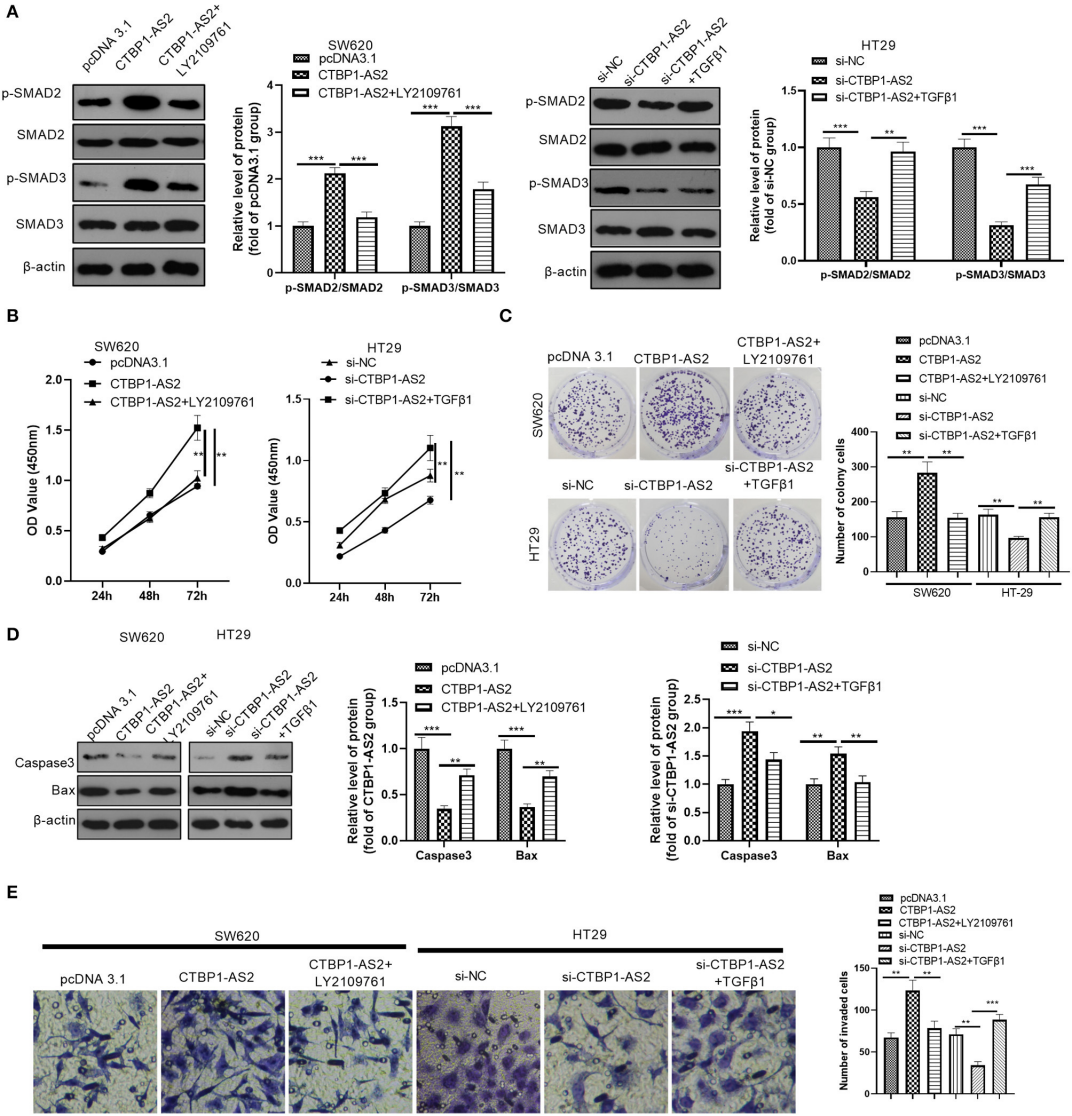
Effect of interference with the TGF-β/SMAD2/3 pathway on CTBP1-AS2 function. **(A)** Use of human recombinant TGF-β1 (100 ng/ml) to activate the TGF-β/SMAD2/3 pathway in HT29 cells transfected with si-CTBP1-AS2, and LY2109761 (5 μM) to inhibit the TGF-β/SMAD2/3 pathway in SW620 cells transfected with CTBP1-AS2 overexpression plasmids for 4 h. Evaluation of the phosphorylation and total protein levels of SMAD2/3 by Western blotting. **(B)** Examination of cell proliferation of SW620 and HT29 cells using CCK-8 assay. **(C)** Detection of cell proliferation of SW620 and HT29 cells using colony formation experiments. **(D)**. Use of Western blotting to measure the expression of apoptosis-related proteins caspase-3 and Bax in tumor cells in SW620 and HT29 cells. **(E)**. Use of the Transwell method to measure cell invasion of SW620 and HT29 cells. *p < 0.05, **p < 0.01, ***p < 0.001. *N* = 3. Abbreviations: CCK-8, cell counting kit-8; TGF-β/SMAD2/3, transforming growth factor-β/small mothers against decapentaplegic 2/3.

Aiming at further exploration on the effect of TGF-β1/SMAD2/3 pathway in the regulation of CTBP1-AS2, we used human recombinant TGF-β1 to activate the TGF-β1/SMAD2/3 pathway and LY2109761 to inhibit the TGF-β1/SMAD2/3 pathway. It was found that LY2109761 inactivated the TGF-β1/SMAD2/3 pathway mediated by CTBP1-AS2, while TGF-β1 treatment enhanced SMAD2/3 phosphorylation (Figure 4A). Then, we examined the CRC cell proliferation, apoptosis, and invasion. The results demonstrated that, compared with the CTBP1-AS2 group, restraining TGF-β1/SMAD2/3 pathway reduced CRC cell proliferation (Figures 4B,C), enhanced the cell apoptosis (Figure 4D), and inhibited the CRC cell invasion (Figure 4E). Compared with the si-CTBP1-AS2 group, the activation of the TGF-β1/SMAD2/3 pathway accelerated CRC cell proliferation (Figures 4B,C) and invasion (Figure 4E), while reducing the cell apoptosis (Figure 4D). Thus, CTBP1-AS2 regulates the CRC cell proliferation, apoptosis, and invasion in a TGF-β1/SMAD2/3-dependent pathway.

### miR-93-5p Was a Target for CTBP1-AS2 and TGF-β1

**Figure 5:**
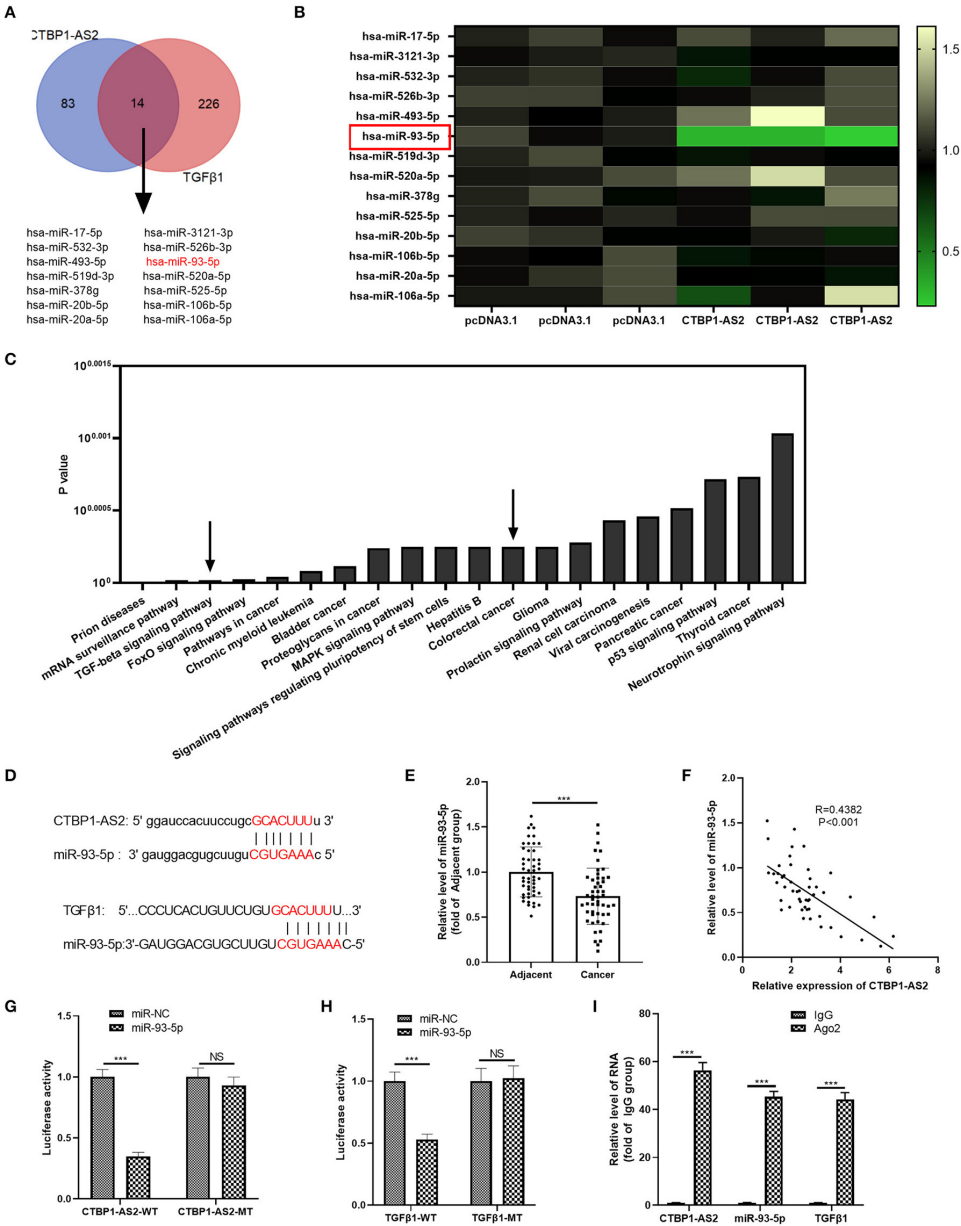
miR-93-5p as a potential target for CTBP1-AS2 and TGF-β1. (A) Prediction of miRNAs targeting CTBP1-AS2 by Starbase (http://starbase.sysu.edu.cn/), prediction of miRNAs targeting TGF-β1 by TargetScan (http://www.targetscan.org/vert_72/), use of Venn diagram to analyze the common miRNA of the two. Fourteen potential miRNAs were found. **(B)** Detection of the 14 miRNAs in SW620 cells transfected with CTBP1-AS2 or pcDNA3.1 by performing RT-PCR. **(C)** Analysis of the signal pathways involved in miR-93-5p through mirPath version 3. **(D)** Base pairing sites of miR-93-5p with CTBP1-AS2 and TGF-βTGF-β1 are shown. **(E)** Detection of miR-93-5p expression in CRC tissues and the adjacent normal tissues by RT-PCR. **(F)** Analysis of the correlation between miR-93-5p and CTBP1-AS2 in CRC tissues by Pearson's linear regression. **(G,H)** Use of dual-luciferase activity experiments to verify the binding relationship of miR-93-5p to CTBP1-AS2 **(G)** and TGF-β1 **(H)**. **(I)** Use of RIPA lysis buffer to verify the relationship of miR-93-5p, CTBP1-AS2, and TGF-β1. Performing the RT-PCR to detect the levels of CTBP1-AS2, miR-93-5p, and TGF-β1 in RIPA precipitates. ***p < 0.001. *N* = 3. CRC, colorectal cancer; miRNA, microRNA; NS, not significant; RIPA, radioimmunoprecipitation assay; RT, reverse transcription; TGF-β, transforming growth factor-β.

Inspired by the regulatory axis of lncRNA–miRNA–mRNA, we attempted to explore the regulatory molecules between CTBP1-AS2 and TGF-β1. The results demonstrated that 14 miRNAs were the potential targets (Figure 5A). Next, we performed RT-PCR to detect those miRNAs in SW620 cells with upregulated CTBP1-AS2. It was found that miR-93-5p was markedly downregulated in SW620 cells (compared with pcDNA3.1 group) (Figure 5B), suggesting that miR-93-5p is a potentially important target. Through mirPath version 3 (http://snf-515788.vm.okeanos.grnet.gr/index.php?R=mirpath), we found that miR-93-5p participated in the TGF-β signaling pathway and in the progression of CRC (Figure 5C). The binding sites between miR-93-5p and CTBP1-AS2, miR-93-5p, and TGF-β1 are shown in Figure 5D. Then, we detected miR-93-5p expression in CRC tissues, and the statistics showed that it was downregulated compared with healthy adjacent CRC tissues (Figure 5E) and had a negative correlation with CTBP1-AS2 (Figure 5F). In addition, the online databases Starbase and Kaplan–Meier plotter both indicated that lower level of miR-93-5p was associated with worse survival of patients with CRC (*p* = 0.063) (Supplementary Figures 2A,B). Interestingly, the levels of miR-93-5p and TGF-β1 were significantly negatively correlated in COAD and READ (Supplementary Figures 3A,B). Besides, through the dual-luciferase activity assay, we found that miR-93-5p mimics remarkably inhibited the dual-luciferase activity of SW620 cells transfected with CTBP1-AS2-WT and TGF-β1-WT, but had no significant effect on CTBP1-AS2-MT and TGF-β1-MT (Figures 5G,H). Besides, experimental results regarding RIPA lysis buffer illustrated that, compared with IgG group, the enrichment of CTBP1-AS2, miR-93-5p, and TGF-β1 in Ago2 group increased significantly (Figure 5I). In summary, the data mentioned above indicate that miR-93-5p is a key regulatory molecule intermediate between CTBP1-AS2 and TGF-β1.

### The Effect of CTBP1-AS2/miR-93-5p on CRC Progression

**Figure 6:**
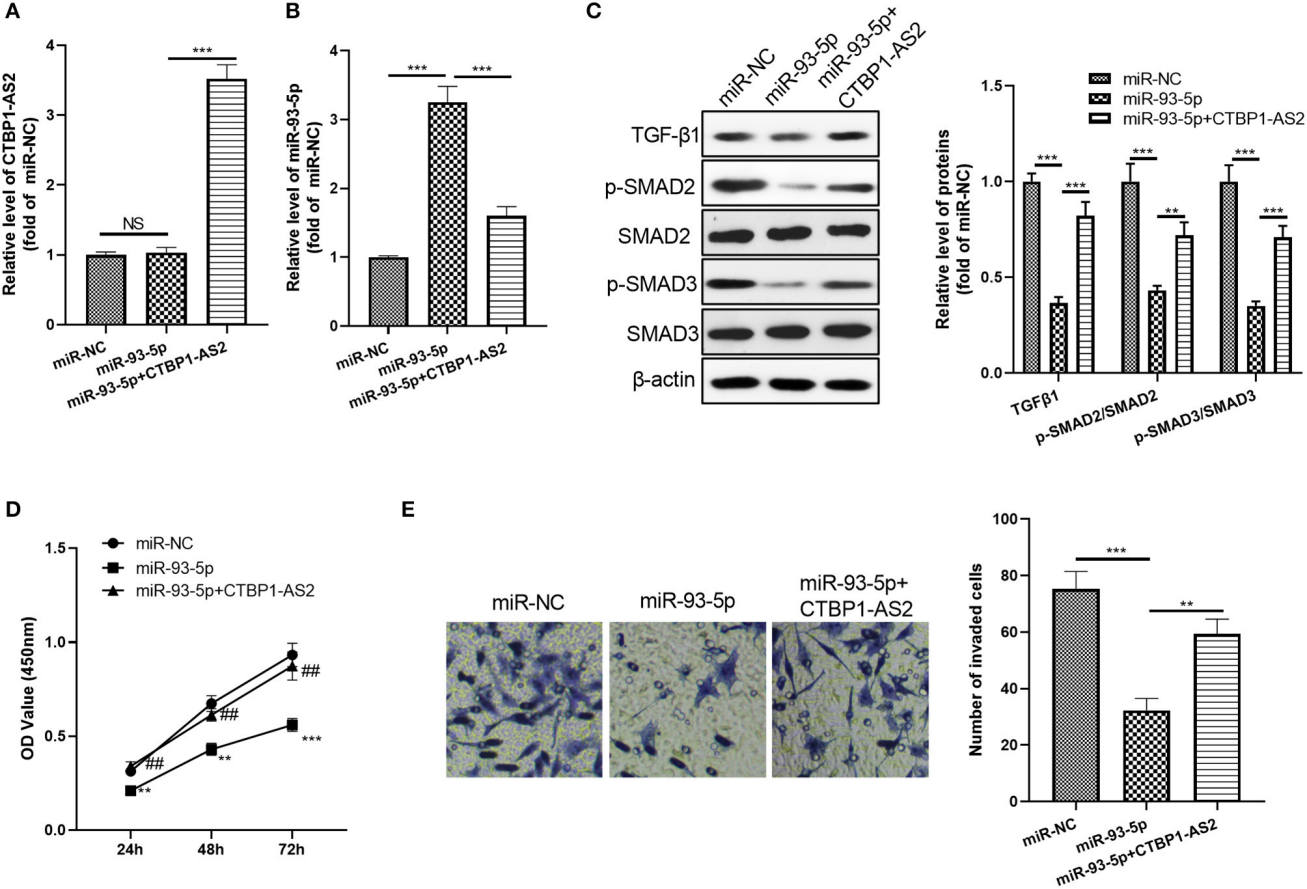
Effect of CTBP1-AS2/miR-93-5p on CRC proliferation and invasion. **(A,B)** Construction of a cell model of miR-93-5p overexpression in SW620 cells, and adding CTBP1-AS2 overexpression plasmid to investigate the interaction of CTBP1-AS2-miR-93-5p axis in CRC. Use of RT-PCR to measure the levels of CTBP1-AS2 **(A)** and miR-93-5p **(B)**. **(C)** Detection of the levels of TGF-β1, SMAD2, and SMAD3 in the cells by using Western blotting. **(D)** Use of CCK-8 to examine the cell proliferation. **p < 0.01, ***p < 0.001 vs. miR-NC group, ##p <0.01 vs. miR-93-5p group. **(E)** Use of the Transwell method to detect cell invasion. **p < 0.01, ***p < 0.001. *N* = 3. CCK-8, cell counting kit-8; CRC, colorectal cancer; NC, negative control; NS, not significant; RT, reverse transcription; TGF-β, transforming growth factor-β.

Considering the expression characteristics of miR-93-5p and CTBP1-AS2 in CRC, we further constructed an overexpressed miR-93-5p cell model and added overexpressed CTBP1-AS2 plasmids to study the interaction of CTBP1-AS2-miR-93-5p in CRC progression. The results showed that CTBP1-AS2 overexpression notably decreased miR-93-5p expression compared with the miR-93-5p group (Figures 6A,B). Moreover, after miR-93-5p was overexpressed, the TGF-β/SMAD2/3 pathway was significantly inhibited, while supplementation of CTBP1-AS2 upregulated the expression of TGF-β/SMAD2/3 (Figure 6C). Through CCK-8 and Transwell experiments, we discovered that overexpressed miR-93-5p considerably inhibited CRC cell proliferation and invasion, while these effects were reversed by CTBP1-AS2 overexpression (Figures 6D,E). Therefore, the CTBPA-AS2 expression promoted the proliferation and invasion *via* inhibiting miR-93-5p.

### CTBP1-AS2 Aggravated the Growth and Metastasis of CRC Cells *in vivo*

**Figure 7:**
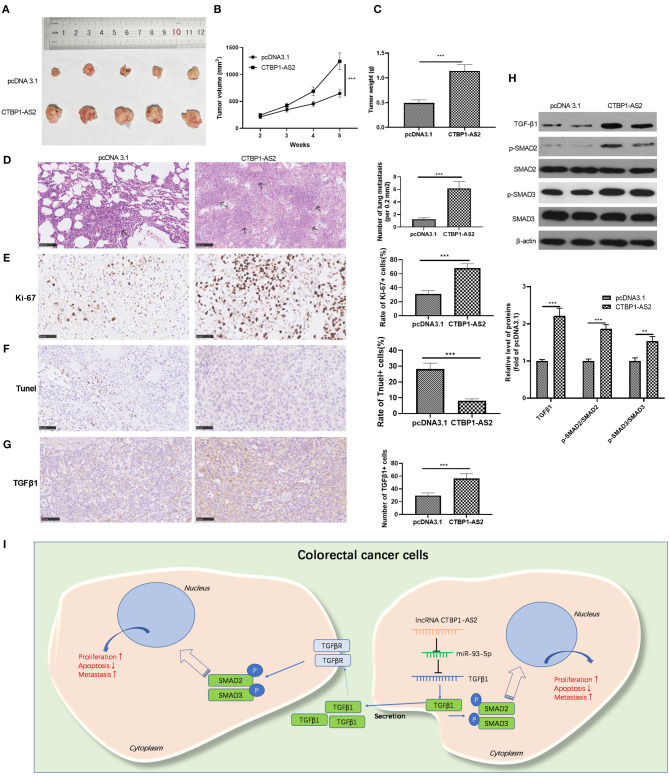
Effect of CTBP1-AS2/miR-93-5p on CRC proliferation and invasion. **(A,B)** Construction of a cell model of miR-93-5p overexpression in SW620 cells, and adding CTBP1-AS2 overexpression plasmid to investigate the interaction of CTBP1-AS2-miR-93-5p axis in CRC. Use of RT-PCR to measure the levels of CTBP1-AS2 **(A)** and miR-93-5p **(B)**. **(C)** Detection of the levels of TGF-β1, SMAD2, and SMAD3 in the cells by using Western blotting. **(D)** Use of CCK-8 to examine the cell proliferation. **p < 0.01, ***p < 0.001 vs. miR-NC group, ##p <0.01 vs. miR-93-5p group. **(E)** Use of the Transwell method to detect cell invasion. *p > 0.05, **p < 0.01, ***p < 0.001. *N* = 3. CCK-8, cell counting kit-8; CRC, colorectal cancer; NC, negative control; NS, not significant; RT, reverse transcription; TGF-β, transforming growth factor-β.

We explored the effect of CTBP1-AS2 expression on the growth of CRC cells in tumors *in vivo*. The results represented that the overexpression of CTBP1-AS2 markedly accelerated the growth of SW620 cells (Figures 7A–C). In addition, CTBP1-AS2 upregulation enhanced the lung metastasis of SW620 cells (Figure 7D), increased the rate of Ki-67-positive cells while reducing the rate of TUNEL-positive cells (Figures 7E,F). We also measured TGF-β1 and SMAD2/3 expression in tumor tissues by using immunohistochemistry or Western blotting. The results illustrated that upregulation of CTBP1-AS2 notably promoted TGF-β1 expression and the phosphorylation levels of SMAD2 and SMAD3 (Figures 7G,H). Collectively, in CRC, upregulated CTBP1-AS2 promotes TGF-β expression by inhibiting miR-93-5p. Meanwhile, inside the cells, upregulated TGF-β activates SMAD2/3 to promote CRC cell proliferation and invasion and inhibits cell apoptosis. Furthermore, intracellular TGF-β can also be exocrine to adjacent cells to accelerate its growth and metastasis (Figure 7I).

## Discussion

In this study, we investigated the mechanism of CTBP1-AS2 in regulating CRC proliferation and metastasis through *in vitro* and *in vivo* experiments. Our results suggested that CTBP1-AS2 can not only be used as a prognostic and diagnostic indicator of CRC, but can also promote CRC development by regulating the miR-93-5p/TGF-β/SMAD2/3 axis (Figure 7I).

In the course of CRC progression, unrestricted proliferation and metastasis are the core factors that contribute to the worse survival of patients with CRC. A variety of clinical drugs exert their effects by inhibiting the proliferation of tumor cells (15, 16). Besides, for patients with CRC with localized lesions, the 5-year survival rate is as high as 90%. However, once local metastasis and distant metastasis of CRC occur, the 5-year survival rate of patients with CRC decreases by 70% and 15–20%, respectively (17). Therefore, it is very important to develop indicators for the early diagnosis of CRC and implement early treatment. In recent decades, accumulating lncRNAs have been found to be abnormally expressed in CRC and can be used as potential therapeutic targets. Taking lncRNA CRNDE as an example, it is upregulated in CRC tissues and promotes the Wnt/β-catenin pathway by inhibiting miR-181a-5p, thus promoting the proliferation and chemotherapeutic resistance of CRC (18). Moreover, lncRNA BANCR upregulation promoted the proliferation, invasion, and adriamycin resistance of CRC (19). As a novel lncRNA, CTBP1-AS2 was found to regulate the ardiomyocyte hypertrophy in previous studies (8). In particular, CTBP1-AS2 is a potential prognostic indicator for papillary thyroid cancer (20). Here, we initially found that high levels of CTBP1-AS2 can be used as a molecular marker for worse survival of patients with CRC, and its overexpression accelerates CRC cell proliferation and invasion and inhibits cell apoptosis. This fact suggests that CTBP1-AS2 has an oncogenic effect in CRC.

In addition to lncRNAs, a variety of miRNAs have also been found to be extensively involved in CRC progression. By regulating the expression of tumor-related genes, miRNA makes a significant contribution to the regulation of cell proliferation, apoptosis, metastasis, stem cell maintenance, angiogenesis, and chemotherapy resistance of CRC. For example, miR-145-5p (21) and miR-873-5p (22) are downregulated in CRC, which can reduce the progress of CRC by inhibiting cell proliferation, invasion, and epithelial–mesenchymal transformation. Similarly, miR-375-3p exerts an antitumor role by enhancing the sensitivity of CRC to 5-fluorouracil by targeting thymidylate synthase (23). In turn, doxorubicin can upregulate PD-L1 by inhibiting miR-140, a tumor suppressor miRNA (24). Besides, miR-25-3p from tumor cell exosomes promotes CRC development by accelerating angiogenesis (25). These studies fully confirm that miRNA functions in CRC growth diversely, both as a carcinogenic and anticancer molecule. In previous studies, miR-93-5p showed a bidirectional effect in CRC progression, either by inhibiting PD-L1 from weakening CRC cell transfer (11) or by inhibiting cyclin-dependent kinase inhibitor 1A from enhancing the multidrug resistance of CRC cells (26). In the present study, we proved that miR-93-5p is a competitive miRNA of CTBP1-AS2 and is limited by the upregulated CTBP1-AS2. Furthermore, *in vivo* and *in vitro* experiments confirmed that miR-93-5p upregulation inhibited CRC proliferation and invasion, while the compensation experiments confirmed that CTBP1-AS2 upregulation inhibited miR-93-5p and promoted tumor progression, indicating that miR-93-5p was a negative regulator of CRC progression.

As a classic cytokine, TGF-β plays an essential role in inflammation, tissue repair, and embryonic development by interacting with TGF receptors on the cell surface (27, 28). In recent decades, TGF-β has been proved to have an essential regulatory effect on the growth, differentiation, and immune function of tumor cells. In healthy cells and early cancer cells, TGF-β pathway functions in tumor inhibition, including cell cycle arrest and apoptosis. However, its activation in advanced tumors accelerates tumor development, including metastasis and drug resistance. Therefore, it is an important target for tumor immunotherapy (29, 30). In addition, SMAD2/3, as an intracellular signal transduction molecule in the TGF-β pathway, is phosphorylated after the action of TGF-β, leading to tumor formation (31, 32). Interestingly, miRNA regulates tumor development by regulating the TGF-β pathway. Upregulation of miR-582-3p and miR-582-5p has been found to limit bone metastasis of prostate cancer by inhibiting TGF-β signaling (33). Here, we found that CTBP1-AS2 upregulated the intracellular and extracellular expression of TGF-β1 and its downstream phosphorylation of SMAD2/3. Interference with TGF-β and SMAD2/3 significantly affected CTBP1-AS2-mediated CRC progression. Furthermore, we found that miR-93-5p is a downstream molecule of CTBP1-AS2 and targets the 3′-UTR of TGF-β. Functionally, overexpressed miR-93-5p exerts an anticancer effect by inhibiting the TGF-β/SMAD2/3 pathway.

## Conclusion

This study explored the effect of the CTBP1-AS2-miR-93-5p-TGF-β/SMAD2/3 axis in CRC development. Our results indicate that CTBP1-AS2 activates the TGF-β/SMAD2/3 pathway by inhibiting miR-93-5p, resulting in increased CRC cell proliferation and invasion and decreased apoptosis. To sum up, this study provides a new reference for CRC progress. However, the diagnostic value of CTBP1-AS2 in CRC still needs to be verified in more research samples.

## Data Availability Statement

The datasets presented in this study can be found in online repositories. The names of the repository/repositories and accession number(s) can be found in the article/Supplementary Material.

## Ethics Statement

The studies involving human participants were reviewed and approved by Ethics Review Board of The Sixth Affiliated Hospital of Sun Yat-sen University. The patients/participants provided their written informed consent to participate in this study. The animal study was reviewed and approved by The Animal Care and Use Committees of Yantai Affiliated Hospital of Binzhou Medical University. Written informed consent was obtained from the individual(s) for the publication of any potentially identifiable images or data included in this article.

## Author Contributions

QL, WY (2nd author), and SC: conceived and designed the experiments. ZJ and WY (11th author): performed the experiments. YL, TZ, and ML: statistical analysis. ZJ: wrote the paper. All authors read and approved the final manuscript.

## Conflict of Interest

The authors declare that the research was conducted in the absence of any commercial or financial relationships that could be construed as a potential conflict of interest.

## Supplementary Material

Supplementary Figure 1 **(A)**. CTBP1-AS2 expression in human tissues. **(B)**. CTBP1-AS2 expression in 20 human tumor tissues and adjacent healthy tissues.

Supplementary Figure 2 **(A)**. Relationship between miR-93-5p levels and overall COAD survival. **(B)**. Relationship between miR-93 levels and overall READ survival.

Supplementary Figure 3 **(A)**. Correlation of miR-93-5p levels with TGFβ1 in COAD. **(B)**. Correlation of miR-93-5p levels with TGFβ1 in READ.
