# Supplementary figures and images for: Downregulating Long Non-coding RNAs CTBP1-AS2 Inhibits Colorectal Cancer Development by Modulating the miR-93-5p/TGF-β/SMAD2/3 Pathway
Source: Front Oncol. 2021 Apr 14;11:626620. doi: 10.3389/fonc.2021.626620 (PMC8079788; doi:10.3389/fonc.2021.626620)

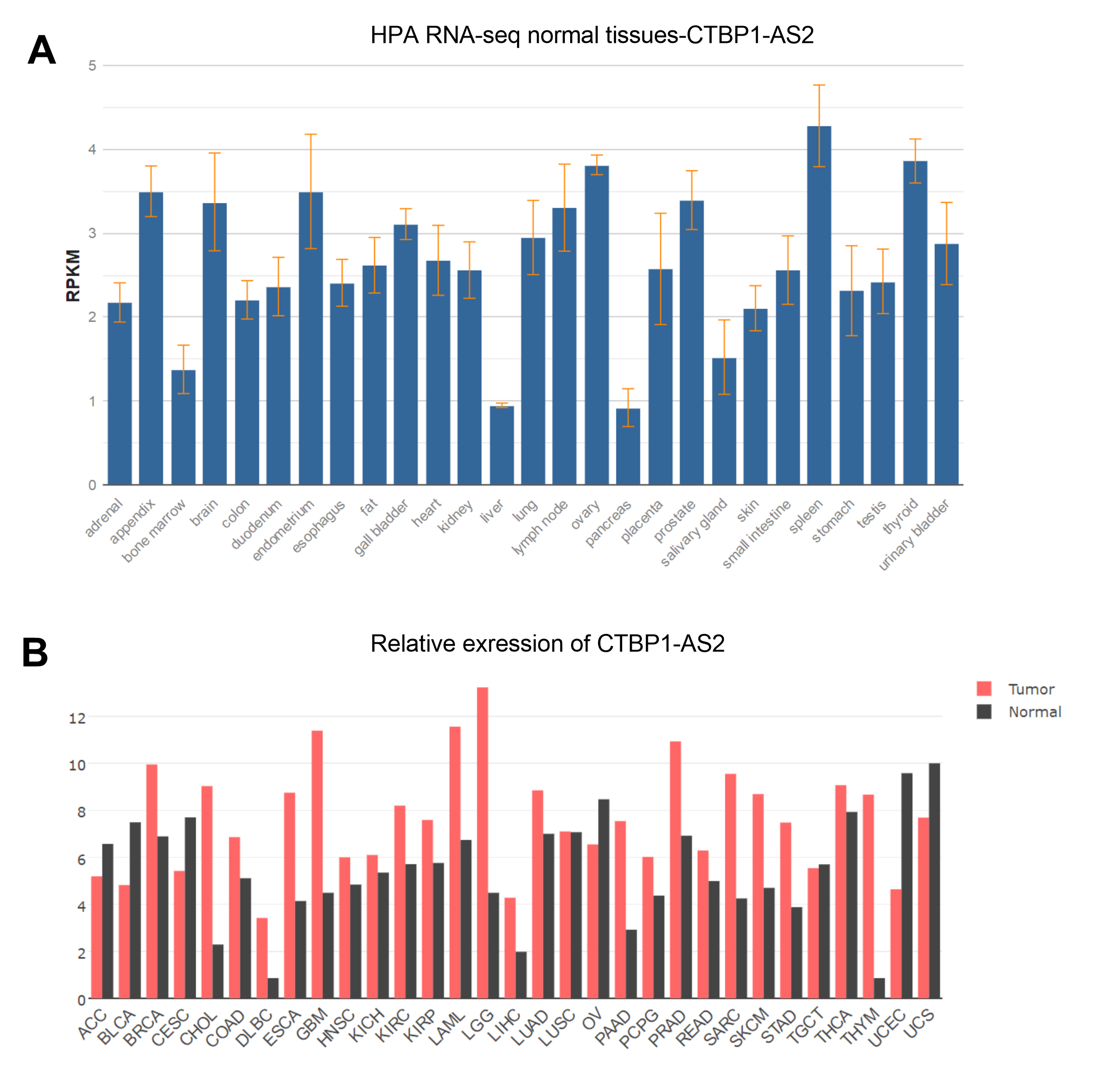

Supplement: Supplementary Figure 1 — (A). CTBP1-AS2 expression in human tissues. (B). CTBP1-AS2 expression in 20 human tumor tissues and adjacent healthy tissues. [file Image_1.TIF]

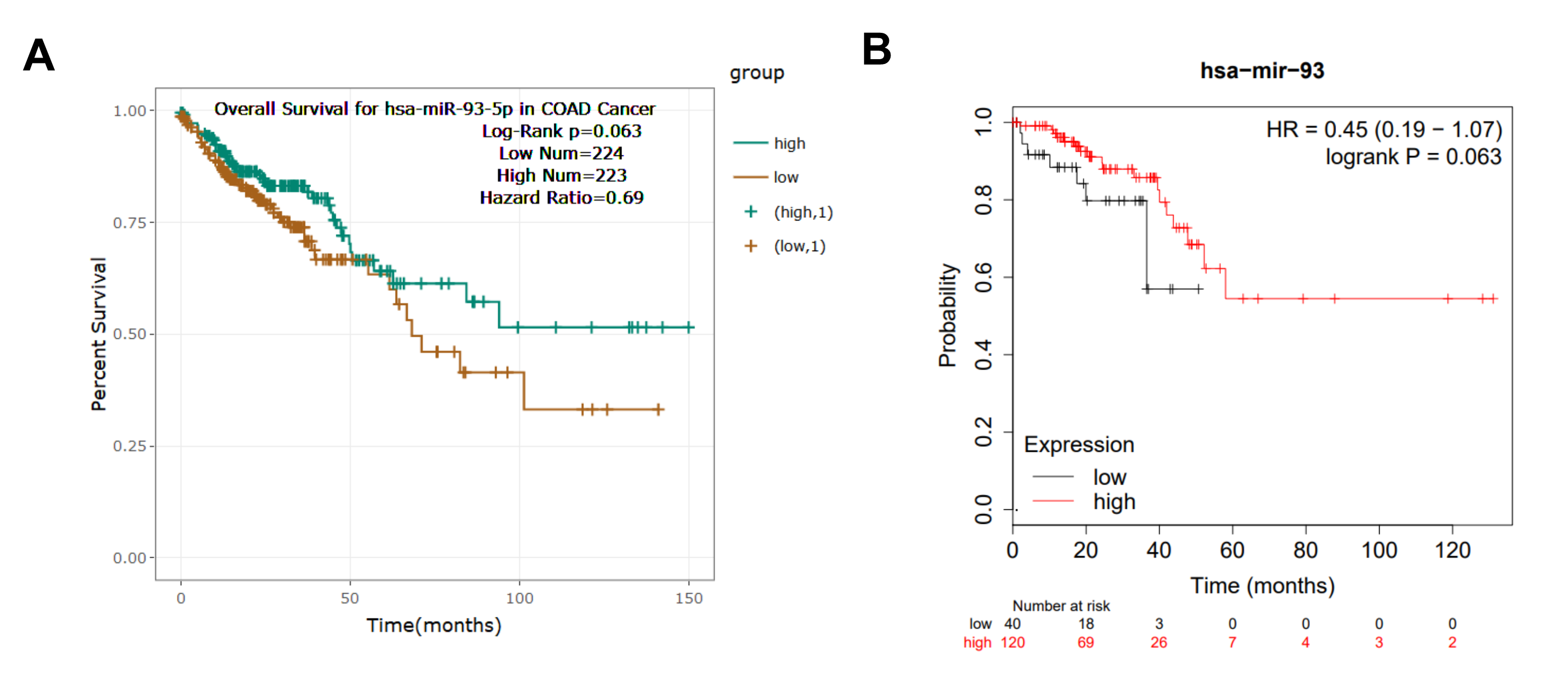

Supplement: Supplementary Figure 2 — (A). Relationship between miR-93-5p levels and overall COAD survival. (B). Relationship between miR-93 levels and overall READ survival. [file Image_2.TIF]

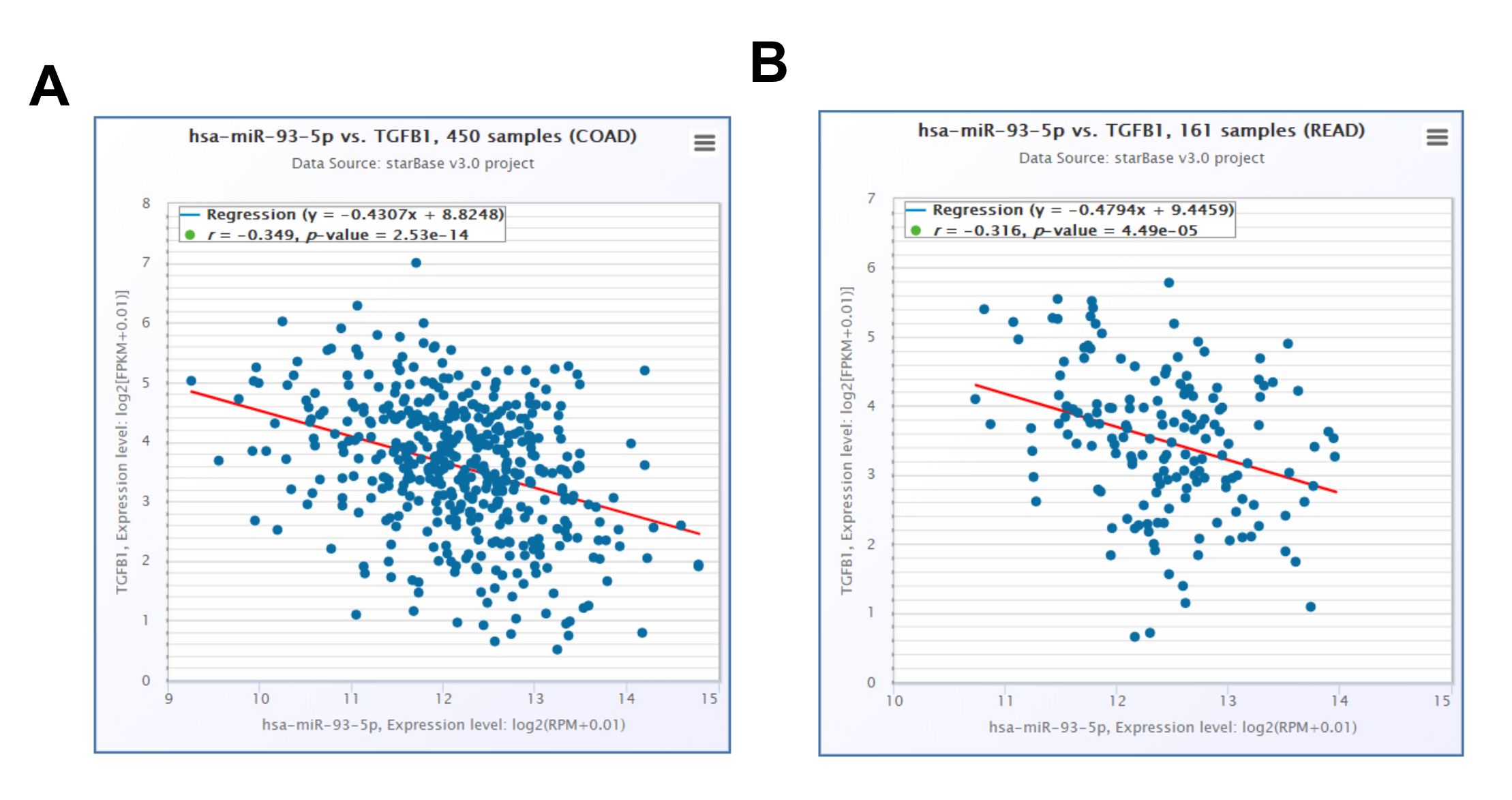

Supplement: Supplementary Figure 3 — (A). Correlation of miR-93-5p levels with TGFβ1 in COAD. (B). Correlation of miR-93-5p levels with TGFβ1 in READ. [file Image_3.TIF]
